# Prognostic role of circulating cytokines and inflammation indexes for avelumab maintenance in metastatic urothelial carcinoma

**DOI:** 10.3389/fimmu.2024.1401214

**Published:** 2024-05-10

**Authors:** Brigida Anna Maiorano, Giovanni Schinzari, Carmine Carbone, Geny Piro, Ernesto Rossi, Massimo Di Maio, Annamaria Di Giacomo, Evaristo Maiello

**Affiliations:** ^1^Oncology Unit, IRCCS Casa Sollievo Della Sofferenza, San Giovanni Rotondo, Italy; ^2^Department of Medical Oncology, IRCCS San Raffaele Hospital, Milan, Italy; ^3^Medical Oncology, Università Cattolica del Sacro Cuore, Rome, Italy; ^4^Medical Oncology, Fondazione Policlinico Universitario Agostino Gemelli Istituti di Ricovero e Cura a Carattere Scientifico (IRCCS), Rome, Italy; ^5^Department of Oncology, University of Turin, Turin, Italy; ^6^Center for Immuno-Oncology, University Hospital of Siena, Siena, Italy

**Keywords:** urothelial carcinoma, avelumab, cytokines, NLR, biomarkers, prognostic, inflammatory indexes

## Abstract

**Background:**

Avelumab maintenance after first-line platinum-based chemotherapy represents a cornerstone for the treatment of metastatic urothelial carcinoma (mUC). However, identifying prognostic biomarkers is paramount for optimizing patients’ benefits while minimizing toxicity. Cytokines represent circulating mediators of the complex interaction between cancer, the immune system, and inflammation. Inflammation, a hallmark of cancer, can be expressed by circulating factors. In different tumor subtypes, peripheral blood biomarkers, such as circulating cytokines, and systemic inflammatory indexes, have been addressed as potential prognostic factors for immune checkpoint inhibitors. However, their role in mUC still needs to be determined.

**Methods:**

Between February 2021 and April 2023, we prospectively collected plasma cytokines and inflammation indexes in 28 patients with mUC before starting avelumab as first-line maintenance. The primary endpoint was the relationship between baseline cytokines and inflammatory indexes with the clinical benefit (CB), defined as the number of Responders. Secondary endpoints included the correlation of baseline cytokines and inflammatory indexes with progression-free survival (PFS), overall survival (OS), and the number and grade of immune-related adverse events.

**Results:**

High pre-treatment levels of interferon (IFN)-γ and interleukin (IL)-2, and low levels of IL-6, IL-8, neutrophil-to-lymphocyte ratio (NLR), lymphocyte-to-monocyte ratio (LMR), and systemic-inflammation index (SII) were associated with clinical benefit and longer survival. In the multivariate analysis, low IL-8, NLR, and SII levels maintained a positive prognostic value for OS.

**Conclusion:**

Our data suggest that, in mUC patients receiving avelumab, pre-treatment levels of plasma cytokines and inflammatory indexes may serve as potential prognostic biomarkers for response and efficacy. In particular, patients with signs of pre-therapeutic inflammation showed a significantly lower response and survival to avelumab. On the contrary, low systemic inflammation and high levels of cytokines characterized responders and longer survivors.

## 1 Introduction

Urothelial carcinoma (UC) is counted among the ten most common cancer subtypes, representing about 3% of tumors, and the thirteenth leading cause of cancer-related death worldwide (1). In the last decade, immune checkpoint inhibitors (ICIs) entered the path of metastatic UC (mUC) therapy, starting from the platinum-progressing setting (2). Starting from 2020, avelumab - an anti-Programmed Death-Ligand 1 (PD-L1) agent, was approved by the Food and Drug Administration (FDA) and the European Medical Agency (EMA) as a maintenance treatment after at least disease stability with platinum-based chemotherapy, based on the JAVELIN Bladder 100 trial (3–5). Despite the considerable advantage in survival and response rates, a group of mUC patients do not benefit from avelumab developing, on the contrary, a rapidly progressive disease with poor outcomes. There is indeed a lack of prognostic and predictive factors for avelumab.

Cytokines are pleiotropic regulators of the host immune activity through the recruitment of immune cells in the tumor microenvironment (TME) produced by immune cells themselves, endothelium, and tumor cells. Several cytokines have been studied to be involved in UC development and spread. Yet, in other tumor types, the prognostic role of baseline circulating cytokines for ICI therapy has been inquired. However, in mUC, most data regard the negative prognostic role of interleukin (IL)-8 for response and survival after ICIs, whereas the role of multiple cytokines as potential biomarkers for response and survival is still controversial (6–8). Besides cytokines production and interaction, immune cells are also involved in inflammation, one of the hallmarks of cancer. Therefore, some inflammatory indexes, such as the neutrophil-lo-lymphocyte ratio (NLR), could reflect the anti-tumor immune response and inflammatory status, and have been correlated with response and survival after ICIs already in other tumor subtypes (9–19).

However, prospective data regarding the impact of systemic inflammation and cytokines profiles on ICIs efficacy in mUC patients are lacking (20, 21). In this scenario, we have investigated the role of baseline expression of circulating cytokines and systemic inflammation indexes as prognostic biomarkers of response to avelumab maintenance in mUC patients.

## 2 Materials and methods

### 2.1 Study subjects

From February 2021 to April 2023, we conducted a prospective study enrolling mUC patients treated with avelumab in two Italian centers. The study was conducted following the ethical principles of the Declaration of Helsinki. Patients were eligible in case of 1) histologically confirmed unresectable locally advanced or metastatic UC; 2) candidates for first-line maintenance with avelumab; 3) at least one measurable lesion at baseline radiological evaluations (computed tomography [CT] scan or magnetic resonance imaging [MRI]); 4) 18 years of age or older; 5) able to sign an informed consent. Patients not eligible for ICIs or treated with ICIs in the adjuvant setting were excluded. The included subject would have been treated with avelumab per clinical practice until progression or unacceptable toxicity occurrence and followed up for 12 months after progression or until death.

### 2.2 Plasma protein detection assay

Plasma samples from included patients were collected prospectively at baseline (1-7 days before avelumab start) in Ethylenediamine tetraacetic acid (EDTA) vacutainer tubes (around 10 mL blood) and processed within 3 hours of collection. Clarified plasma samples were subsequently stored at -80°C.

Protein detection was performed with Luminex xMAP technology using the bead-based R&D human cytokine assay according to the manufacturer’s instructions. We chose a panel of 25 cytokines involved in regulating the identity and function of T-cells and other elements within the TME (22, 23). All samples were analyzed for IL-1β, IL-2, IL-4, IL-5, IL-6, IL-7, IL-8, IL-10, IL-15, IL-17, IL-12/p70, IL-1 Receptor antagonist (IL-1RA), eotaxin/chemokine-(C-C motif)-ligand-11 (CCL-11), granulocyte-colony stimulating factor (G-CSF), granulocyte-macrophage colony-stimulating factor (GM-CSF), interferon (IFN)-γ, monocyte chemotactic protein 1 (MCP-1)/CCL-2, macrophage inflammatory protein 1α (MIP-1α)/CCL-3, MIP-1β/CCL-4, RANTES/CCL-5, chemokine interferon-γ inducible protein 10 (CXCL10), vascular endothelial growth factor (VEGF), platelet-derived growth factor (PDGF), Fibroblast growth factor-2 (FGF2) and tumor necrosis factor (TNF)-α. Plasma samples were centrifuged at 10,000 g at 4°C for 10 minutes, diluted four-fold, and run in duplicate. A minimum of 50 beads per analyte was acquired. Median fluorescence intensities were collected on a Luminex-200 system (Luminex, Bio-Rad) using Bio-Plex Manager software version 6.1 (Bio-Rad). Standard curves for each cytokine were generated using the premixed lyophilized standards provided in the kits. Serial 4-fold dilutions of the standards were run. Cytokine concentrations in samples were determined from the standard curve using a 5-point regression to transform mean fluorescence intensities into concentrations. Each sample was run in duplicate, and the average of the duplicates was used as the measured concentration.

### 2.3 Examined laboratory values

Neutrophil-, lymphocyte-, monocyte-, and platelet counts were recorded from laboratory values before the first cycle of avelumab (range 1-7 days). We subsequently calculated the following indexes:

- Neutrophil-to-lymphocyte ratio (NLR): absolute neutrophil count divided by absolute lymphocyte count;
- Platelet-to-lymphocyte ratio (PLR): absolute platelet count divided by absolute lymphocyte count;
- Lymphocyte-to-monocyte ratio (LMR): absolute lymphocyte count divided by absolute monocyte count;
- Systemic-inflammation-index (SII): absolute platelet count multiplied per NLR.

### 2.4 Clinical outcomes

Response to avelumab was based on radiological evaluation, using the Immune-Response Evaluation Criteria in Solid Tumors (iRECIST) criteria. The radiological evaluations were performed at baseline and every 12 weeks (± 4 weeks).

Patients were classified as responders (Rs) in case of detection of complete response (CR), partial response (PR), or stable disease (SD) at the first evaluation. Patients were defined as not-responders (N-Rs) in case of progressive disease (PD). Patients with a clinical progression or death before a radiological evaluation was possible were defined as N-Rs. In case of suspected pseudo-progression, a second CT scan would have been performed after at least four weeks.

The primary endpoint of our analysis was the relationship between baseline cytokines and inflammatory indexes with the clinical benefit (CB), defined as the number of Rs. Secondary endpoints included the correlation of baseline cytokines and inflammatory indexes with progression-free survival (PFS), overall survival (OS), and the number and grade of immune-related adverse events (irAEs). PFS was defined as the time from the first cycle of avelumab to disease progression or death, whichever occurred first, censored at last follow-up for patients who were alive (without progression). OS was the time that occurred from avelumab start to death, censored at last follow-up for patients who were alive. AEs were measured following the Common Terminology Criteria for Adverse Events (CTCAE) version 4.0.

### 2.5 Statistical analysis

Characteristics of the included patients were summarized using frequencies and proportions for categorical variables and means and ranges for continuous variables. Fischer’s exact test was used to compare categorical variables between Rs and N-Rs, while the Kruskal-Wallis test was used to compare continuous variables.

To establish the cut-off values for baseline cytokines and systemic inflammatory indexes, the Receiver Operating Characteristic (ROC) curve was used, according to response to avelumab (Rs vs. N-Rs), and assuming the null hypothesis (H0) of Area under the curve (AUC)=0.5. Youden’s test was used to determine the threshold values of the ROC curves (24).

OS and PFS rates were estimated with the Kaplan-Meier method and the Mantel-Haenszel method to evaluate the differences between survival curves. Univariable and multivariable analyses for survival were conducted with the Cox proportional hazard-regression models, with Hazard ratios (HR) alongside 95% Confidence intervals (CI) estimation.

P-values <0.05 were considered statistically significant. No correction for multiplicity was applied.

All statistical analyses were performed with the SPSS v.29.0 software and graphs with GraphPad Prism v.9.5.0.

## 3 Results

### 3.1 Baseline characteristics of included patients

From February 2021 to April 2023, 28 patients were recruited. Among them, there were 19 males (67.8%) and 9 females (32.2%). The median age was 68 years (42-84 years). Thirteen patients (46.4%) had Eastern Cooperative Oncology Group Performance Status [ECOG(PS)] 0, 12 (42.8%) had 1, and 3 patients (10.8%) had ECOG(PS) 2. 12 patients (42.8%) were diagnosed in the metastatic stage, 16 (57.2%) relapsed after a previous surgery. The most frequent sites of metastases were visceral sites (75.0%), and 64.3% of patients had three or fewer sites of metastases. PD-L1 status was unknown in most patients (64.2%), as this analysis was not mandatory for avelumab prescription in Italy.

### 3.2 Response and survival outcomes of the overall population

Except for a higher probability of response in patients with equal to less than three metastatic sites (p=0.012), no significant associations between response status or survival and clinical characteristics were found (**Table 1**).

**Table 1:** Characteristics of included patients.

Clinical characteristics	Number of patients	*p* Response	*p* PFS	*p* OS
Total number	28	/	/	/
Sex, *n (%)* Males Females	19 (67.8%) 9 (32.2%)	0.998	0.793	0.721
Median age, *years [range]*	68 [42-84]	0.513	0.143	0.312
ECOG(PS), *n (%)* 0 1 2	13 (46.4%) 12 (42.8%) 3 (10.8%)	0.060	0.628	0.064
Metastatic at diagnosis, *n (%)* Yes No	12 (42.8%) 16 (57.2%)	0.123	0.195	0.593
Sites of metastases, *n (%)* Visceral Bone Nodes Brain	21 (75.0%) 9 (32.2%) 3 (10.8%) 5 (17.8%)	0.296 0.235 0.579 0.095	0.398 0.212 0.541 0.119	0.975 0.069 0.260 0.085
Nr. of metastatic sites, *n (%)* ≤3 >3	18 (64.3%) 10 (35.7%)	0.012	0.808	0.155
Bajorin score, *n (%)* 0 1 2	12 (42.8%) 13 (46.4%) 3 (10.8%)	0.071	0.539	0.321
PD-L1 status, *n (%)* Positive Negative Unknown	7 (25.0%) 3 (10.8%) 18 (64.2%)	0.658	0.262	0.175
Location of primary tumor, *n (%)* Bladder Upper tract	27 (96.4%) 1 (3.6%)	0.495	0.414	0.568

ECOG(PS), ECOG Performance Status; OS, overall survival; PFS, probability of progression-free survival.

At the data cut-off (May 2023), the median follow-up (mFU) was 18.1 months (range: 3.0-32.8 months [mos]). The median treatment exposure to avelumab was 6.0 months (range: 3.0-21.0 mos).

In total, 13 Rs (46.4%) and 15 N-Rs (53.6%) were found. Among them, there were one CR, seven PR, and five SD. Ten patients were still on treatment. The response was significantly associated with PFS (p<0.001) and OS (p=0.005) (**Supplementary Table 1**).

In the overall population, mPFS was 5.8 months (range: 0.3-30.7 mos), with a maximum of 13.3 months in Rs (range: 5.5 mos-not reached [NR]), and 3.0 months in N-Rs (range: 0.3-5.6 mos; p<0.0001).

At the data cut-off, 14 deaths occurred, 2 in the Rs group, and 12 in the N-Rs group (p<0.0001). mOS was 16.0 months in all patients (range: 1.0-30.7 mos), NR in Rs (range: 6.8 mos-NR), and 5.6 months in N-Rs (range: 1.0-14.6 mos). No treatment-related deaths were reported.

### 3.3 Pre-treatment plasma cytokines

Plasma samples were available for all 28 patients. Baseline cytokine levels were detected before avelumab started, and levels were compared between the Rs and N-Rs groups. We found that levels of IL-1β, IL-4, IL-5, IL-7, IL-10, IL-15, IL-17, IL-12p70, eotaxin/CCL-11, G-CSF, GM-CSF, MCP-1/CCL-2, MIP-1α/CCL-3, MIP-1β/CCL-4, RANTES/CCL-5, CXCL10, VEGF, PDGF, FGF2, and TNF-α did not differ between the two groups (p>0.05), while a significant difference was found between Rs and N-Rs in levels of IL-2 (p=0.049), IFN-γ (p=0.035), IL-6 (p=0.034), IL-8 (p=0.014) (**Table 2**).

**Table 2:** Association between baseline values of cytokines and responders or not-responders to avelumab.

Cytokine	p-value	Cytokine	p-value
IL-1β	0.664	CCL-11	0.063
IL-2	0.049*	G-CSF	0.120
IL-4	0.510	GM-CSF	0.117
IL-5	0.098	CCL-2	0.107
IFN-γ	0.035*	CCL-3	0.638
IL-6	0.034*	CCL-4	0.528
IL-7	0.187	CCL-5	0.219
IL-8	0.014*	CXCL10	0.356
IL-10	0.901	VEGF	0.302
IL-15	0.221	PDGF	0.232
IL-17	0.274	FGF2	0.450
IL-12	0.743	TNF- α	0.148
IL-1RA	0.621		

CCL, Chemokine (C-C motif) ligand; CXCL, C-X-C motif chemokine ligand; FGF, fibroblast growth factor; G-CSF, granulocyte-colony stimulating factor; GM-CSF, Granulocyte macrophage colony-stimulating factor; IFN, interferon; IL, interleukin; IL-1RA, interleukin 1-receptor antagonist; PDGF, platelet-derived growth factor; TNF, tumor necrosis factor; VEGF, vascular endothelial growth factor; *, statistically significant values.

**Figure 1:**
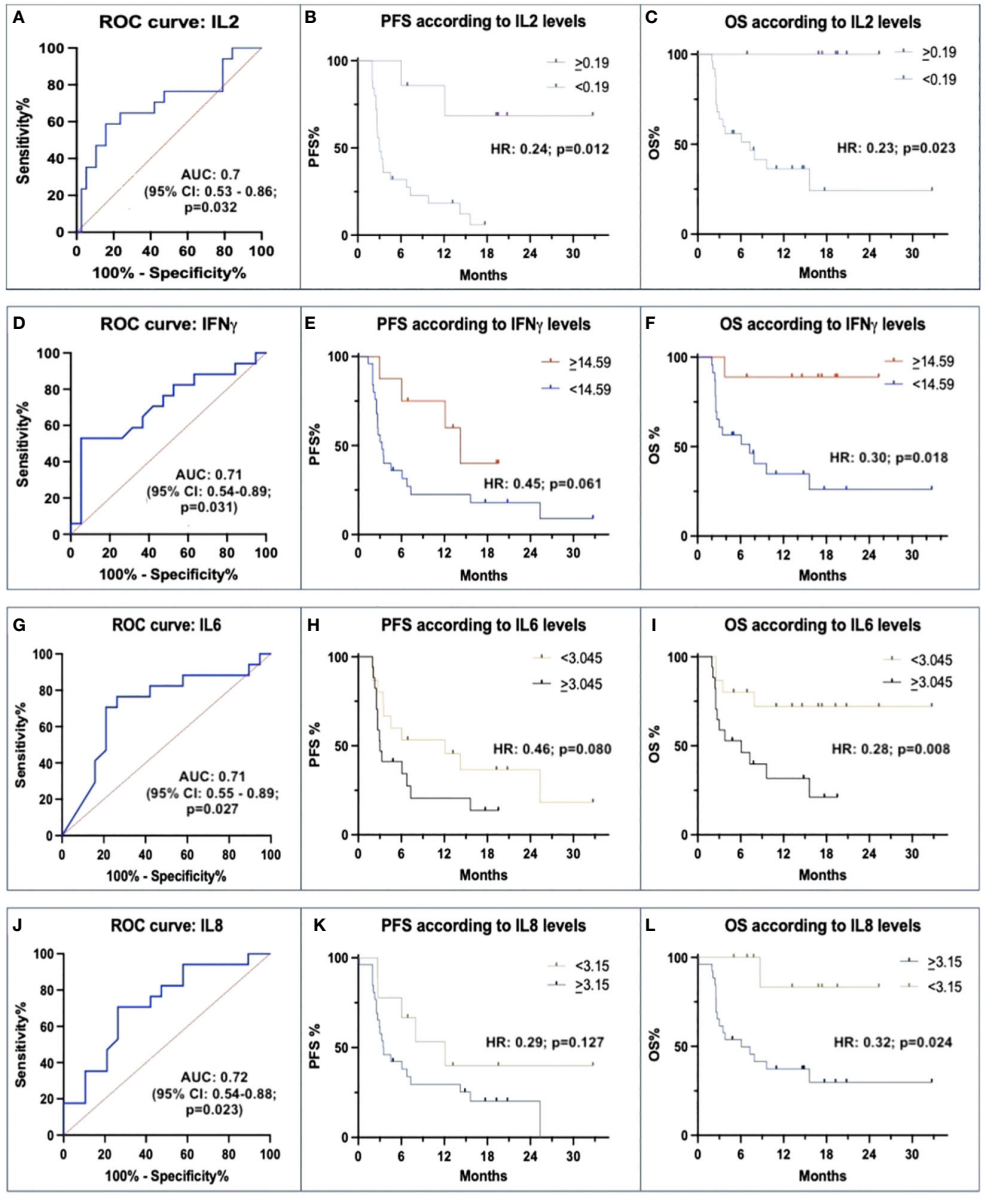
Correlations between baseline IL-2, IFN-γ, IL-6 and IL-8 with avelumab efficacy. **(A, D, G, J)** ROC curves of baseline levels between responders (Rs) and not-responders (N-Rs) show differences for IL-2, IFN-γ, IL-6, and IL-8. **(B, E, H, K)** Kaplan-Meier analyses for PFS show a significant impact of threshold values of IL-2, and not significant for IFN-γ, IL-6, and IL-8. **(C, F, I, L)** Kaplan-Meier analyses for OS show a significant impact of different baseline IL-2, IFN-γ, IL-6, and IL-8 levels.

IL-2 showed a higher expression in Rs than in N-Rs (median 4.17 vs. 0.26 pg/mL). ROC curve with IL-2 levels, using a threshold of 0.19 pg/mL, had a positive predictive value (PPV) of 85% and a negative predictive value (NPV) of 63%. AUC was 0.7 (p=0.032) (**Figure 1A**). HR for PFS was 0.24 (p=0.012), and for OS was 0.23 (p=0.023), with mPFS 6.14 vs. 3.13 months, and mOS NR vs. 7.1 months (**Figures 1B, C**).

Rs were characterized also by higher levels of IFN-γ than N-Rs (median: 28.18 vs. 13.42 pg/mL). ROC curve using a threshold of 14.59 pg/mL showed a PPV of 90% and an NPV of 78%. AUC was 0.71 (p=0.031) (**Figure 1D**). There was a trend for longer mPFS (13.2 vs. 3.3 months; HR 0.45, p=0.061), mOS was NR vs. 7.1 months (HR 0.30, p=0.018) (**Figures 1E, F**).

Rs had lower levels of IL-6 than N-Rs (median: 1.47 vs. 8.08 pg/mL). A 3.045 pg/mL threshold was individuated (PPV 80%, NPV 75%; AUC 0.71, p=0.027) (**Figure 1G**). HR for PFS was 0.46 (p=0.008; median 11.1 vs. 3.03 months), and HR for OS was 0.28 (p=0.008; mOS NR vs. 6.1 months) (**Figures 1H, I**).

Regarding IL-8, a lower expression was detected in Rs than in N-Rs (median: 4.19 vs. 8.08 pg/mL). ROC curve with IL-8 threshold of 3.15 pg/mL, had a PPV of 75% and an NPV of 62%. AUC was 0.72 (p=0.023) (**Figure 1J**). HR for PFS was 0.29 (p=0.127; mPFS 11.1 vs. 3.5 months), and HR for OS was 0.32 (p=0.024; mOS NR vs. 6.0 months) (**Figures 1K, L**).

### 3.4 Pre-therapeutic inflammatory indexes

**Figure 2:**
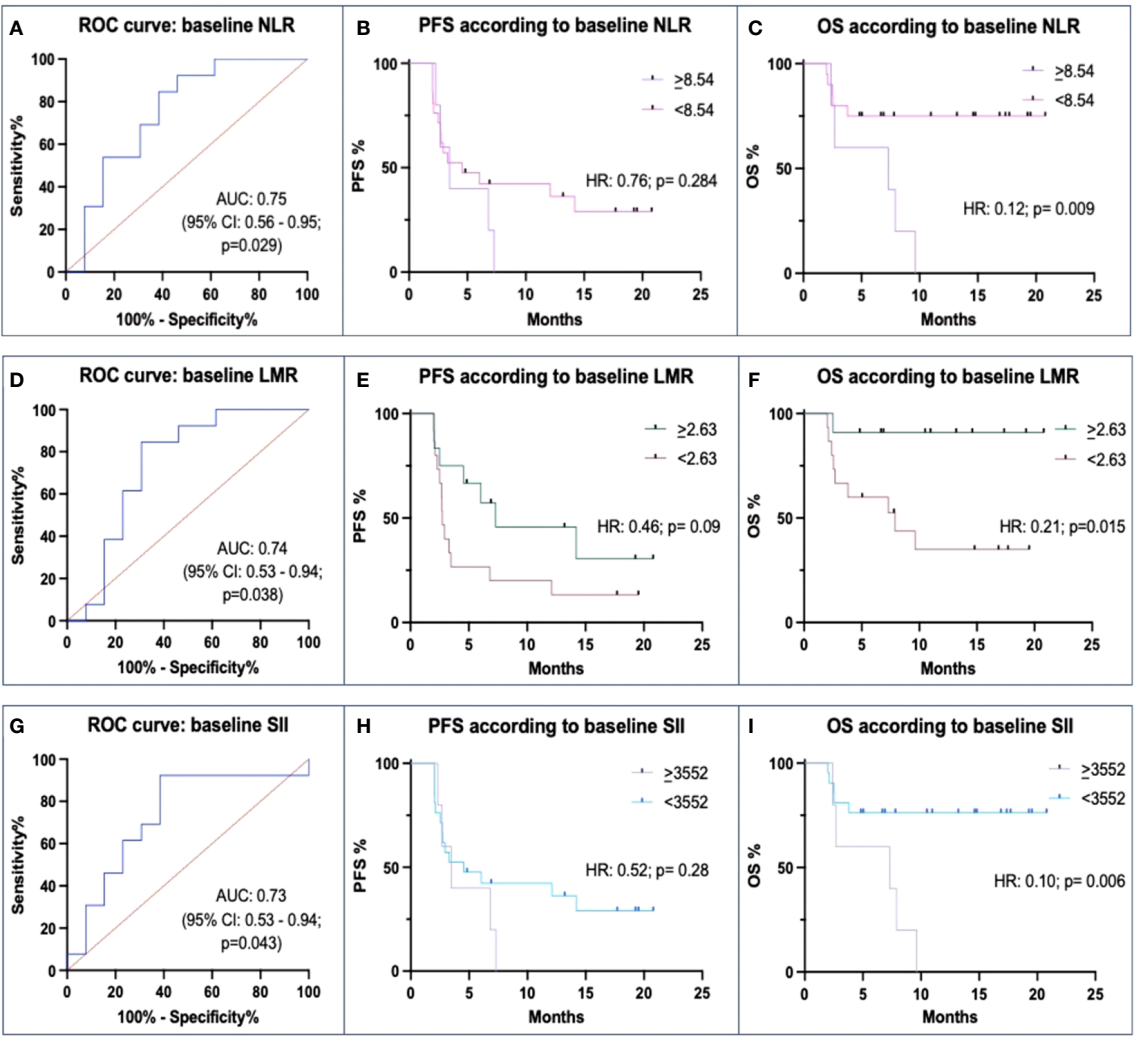
Correlations between baseline Neutrophil-to-lymphocyte ratio (NLR), Lymphocyte-to-monocyte ratio (LMR), Systemic inflammation index (SII) and ICIs efficacy. **(A, D, G)** ROC curves of baseline levels between responders (Rs) and not-responders (N-Rs) show differences for NLR, LMR, and SII. **(B, E, H)** Kaplan-Meier analyses for PFS show a not significant impact of threshold values. **(C, F, I)** Kaplan-Meier analyses for OS show a significant impact of different baseline levels of NLR, LMR, and SII.

We found that pre-treatment NLR values were significantly lower in Rs than in N-Rs (median: 2.72 vs. 6.31; p=0.019). The ROC curve’s AUC was 0.75 (p=0.029) (**Figure 2A**). This had a PPV of 57% and an NPV of 80%. Patients with lower NLR than the threshold value of 8.54 had a numerically higher PFS than patients with NLR ≥8.54 (mPFS: 4.53 vs. 3.47 months; HR: 0.76, p=0.284), and had significantly better OS (HR 0.12, p=0.009; mOS: NR vs. 7.3 months) (**Figures 2B, C**).

There was also a significant difference between Rs and N-Rs regarding LMR, the formers with higher values (median: 3.46 vs. 2.02; p=0.043). In the ROC analysis, AUC was 0.74 (p=0.038), and the threshold value was 2.63 with a PPV of 81%, and an NPV of 73% (**Figure 2D**). Differences in mPFS between patients with higher and lower values (7.3 vs. 2.73 months) were not statistically significant (p=0.09). Instead, mOS (NR vs. 7.9 months) was significantly different between the two groups (p=0.015) (**Figures 2E, F**).

Rs had significantly lower levels of SII values than N-Rs (median: 1099.77 vs. 4607.92; p=0.010). The ROC curve found an AUC of 0.73 (p=0.043) (**Figure 2G**). With a threshold value of 3552, PPV was 57%, and NPV 80%. Patients with low SII levels at baseline had a numerically higher mPFS (4.53 vs. 3.47 mos; p=0.28) and had a longer mOS (NR vs. 7.3 months; p=0.006) (**Figures 2H, I**).

### 3.5 Multivariable analyses of OS

We subsequently integrated the significant values associated with OS (NLR, LMR, SII, IL-2, IFN-γ, IL-6, IL-8) into a multivariate Cox regression analysis, achieving a predictive value for the response of 95%. IL-8 was an independent marker for OS (p=0.019), as well as NLR (p=0.023), and SII (p=0.018) (**Table 3**).

**Table 3:** Multivariate analysis of baseline circulating cytokines and systemic inflammatory indexes and OS.

	HR	95% CI	p-value
NLR	0.33	0.09-0.73	0.023*
LMR	0.197	0.01-1.14	0.120
SII	1.01	1.00-1.02	0.017*
IL-2	0.74	0.48-1.03	0.107
IFN-γ	1.00	0.96-1.05	0.638
IL-6	0.96	0.87-1.06	0.528
IL-8	1.31	1.07-1.7	0.019*

CI, confidence interval; HR, hazard ratio; IL, interleukin; IFN, interferon; LMR, lymphocyte-to-monocyte ratio; NLR, neutrophil-to-lymphocyte ratio; SII, systemic inflammatory index; *, statistically significant values.

### 3.6 Immune-related adverse events assessment

During the whole study period, 6 patients (21.4%) developed G1-2 irAEs (2 hypothyroidism, 1 cutaneous rash, 1 hypercalcemia, 1 hepatic liver enzymes increase, 1 fever). However, no G3-4 irAE leading to treatment discontinuation or death were reported. No association between irAEs, response, and pre-treatment levels of cytokines of systemic inflammation parameters was found (p>0.05) (**Supplementary Table 2**).

## 4 Discussion

Despite a significant improvement in mUC survival, the proper selection of patients most likely to benefit from avelumab maintenance remains an unmet need. To our knowledge, we first prospectively analyzed the baseline levels of a multi-cytokines panel and systemic inflammatory indexes in this setting. We chose a panel of 25 cytokines that regulate the phenotype and function of immune cells within the TME (22, 23). We found that pre-treatment levels of IL-2, IFN-γ, IL-6, IL-8, NLR, LMR, and SII were associated with CB from avelumab and impacted survival. IL-8, NLR, and SII maintained a prognostic role in the multivariate analysis. Our findings support the usefulness of cytokines and inflammatory indexes for selecting mUC patients who likely will respond to avelumab.

IL-8 exerts multiple pro-tumorigenic roles in the TME: it stimulates tumor cell proliferation and spread, favors angiogenesis, and recruits many pro-tumorigenic myeloid inflammatory and immune suppressive cells; moreover, it drives the epithelial-to-mesenchymal transition. In a biomarker analysis of the IMvigor210 trial, high baseline IL-8 was significantly associated with short OS and low overall response rate after atezolizumab both in platinum-progressing and in cis-unfit patients (7). Our findings confirm the negative prognostic role of IL-8 in mUC patients treated with the anti-PD-L1 avelumab. Several ongoing studies investigate the inhibition of IL-8 in combination with an ICI (23, 25, 26).

IFN-γ enhances T-effectors and suppresses Tregs, Myeloid-derived suppressive cells (MDSCs), and pro-angiogenic pathways. After IFN-γ stimulation, Tumor-associated macrophages (TAMs) polarize in M1-like, with antigens-presenting function to lymphocytes and pro-inflammatory cytokines production such as IL-12, IL-1, TNF-α. IFN-γ promotes Th1, CD8^+^, and natural killer (NK) cells and dampens anti-inflammatory cytokines. Therefore, we can speculate that the higher IFN-γ levels are, the more robust immune system activation could be found (27). Gene expression profiles (GEP) analyses have indicated IFN-γ expression as a consistently good predictor of ICIs outcomes in mUC patients (6, 28). In our study, circulating levels of IFN-γ were directly proportional with response probability and OS, with a positive trend for PFS.

On the contrary, we observed a negative association between pre-treatment IL-6 concentrations and CB. The negative prognostic role of IL-6 for ICIs has already emerged in other tumor subtypes (29–31). In our sample, lower levels of IL-6 were associated with response and longer OS. IL-6 is involved in tumor survival and growth (32–34). In the TME, IL-6 recruits immune suppressive mesenchymal stem cells and MDSCs, which reduce CD8^+^ T-cells infiltration and response (31, 32, 35–38). IL-6 suppresses IFN-γ and induces the expression of angiogenic factors, such as VEGF (32, 39). Effectively, IL-6 inhibitors (such as tocilizumab and siltuximab) have shown potential clinical benefit in many cancer subtypes.

IL-2 is central in modulating the innate and adaptive immune systems, as it promotes T-cell expansion, survival, and differentiation after antigen exposure, CD4+, and NK-cells growth and function. Therefore, inhibiting the immune checkpoints may increase IL-2 secretion, enhancing the immune response. IL-2 levels have been associated with better survival after ICIs in other tumor subtypes (40). In a recent meta-analysis of 24 studies with 6,936 ICI-treated cancer patients, IL-2 levels were significantly associated with longer OS, and inversely, IL-6 and IL-8 levels were with shorter OS (41). Also in our study, baseline levels of IL-2 were associated with response and survival.

Regarding systemic inflammation, we found that signs of pre-therapeutic acute inflammation elements were associated with poor response and reduced survival after avelumab. Pre-treatment levels of NLR, SII, and LMR significantly differed between Rs and N-Rs, and predicted response and OS, whereas only higher LMR levels were associated with longer PFS. Our findings align with other studies, from which the most robust data regard the negative prognostic role of NLR (42–44). In mUC, most studies included small cohorts treated with second-line pembrolizumab. Fifty patients with mUC treated by Miyama and colleagues achieved longer PFS and OS, although the results only narrowed the statistical significance (p=0.0056 for PFS, p=0.0054 for OS) (14). In 29 patients treated by Shimizu and colleagues, NLR predicted PFS (16). Moreover, higher NLR after six weeks was associated with OS in 41 Japanese patients treated by Tamura and colleagues (17). Finally, in the retrospective analysis of 121 platinum-refractory patients treated with pembrolizumab by Yamamoto and colleagues, NLR was correlated with OS and ORR (15). Similarly to other tumor types, higher SII levels were negative prognostic factors in mUC patients (45). In a pooled analysis of 9 studies including 7,726 patients, elevated pre-treatment NLR were associated with reduced OS (HR=1.27). Moreover, they were associated with shorter PFS and other characteristics such as advanced tumor stage, size and grade, lymph vascular invasion, nodal invasion, and multifocality (46). The prognostic role of systemic inflammatory indexes has also been investigated with second-line atezolizumab. In the Italian SAUL cohort of 263 patients, SII, PD-L1, and lactate dehydrogenase (LSH) levels were useful to stratify patients’ prognosis and was correlated with OS to second-line atezolizumab (18). In another cohort of 113 patients, elevated pre-treatment levels of NLR, associated with the Bellmunt score, were associated with a shorter OS (19). Besides the strong association with response and survival, an added value of systemic inflammatory indexes is that they can be calculated from a minimally invasive blood count.

Despite the potential clinical impact of our results, this study has several limitations. Firstly, the small number of patients and the observational design could have introduced selection bias and restricted the generalizability of the results. Secondly, the degree of baseline values of cytokines, as well as the inflammatory parameters, might have been affected by previous chemotherapy. Moreover, serum levels of cytokines are highly variable due to their short half-lives, for example, if compared to peripheral blood mononuclear cells’ half-life (47). Furthermore, multiple timepoint analyses still need to be included to understand the longitudinal evolution of cytokine and inflammatory indexes. It is noteworthy that, besides the values that were prognostic in our cohort, several other circulating factors are directly involved in the modulation of an effective anti-tumor response and, therefore, could exert a prognostic role when ICIs release the brakes for the immune system activation (e.g., IL-10, IL-1, TNF-α): disappointingly, our results were not statistically relevant about these further factors, and from previous studies, no conclusive survival data are retrievable. Still, the prognostic role of cytokines and the selection of multiple circulating factors remain a knot to be unraveled in mUC. The cytokines, assays, and thresholds we chose have not previously been validated for diagnostic use in mUC. We decided to calculate the indexes’ cut-offs from the ROC curves of our cohort due to the high heterogeneity between values found in the literature. Effectively, the threshold values are different compared with the literature (15–19, 42–46). This may be partially due to the small sample size of our study. Therefore, more patients are warranted to obtain more accurate predictive values. Future studies should individuate more accurate cut-offs and integrate them into multi-parameter models, incorporating genomic alterations, immune profiling, and other markers, such as PD-L1 or tumor mutational burden (28, 48). Finally, despite the controversial role of PD-L1, which failed to demonstrate a predictive capability for ICIs response, we could not drive conclusions regarding the association of circulating factors with PD-L1 because, at the time of this analysis, we had no available PD-L1 levels for all the included patients. Indeed, besides cytokines, the expression of other immune parameters, such as PD-L1, or PD-L2, could have influenced the responses of our patients. Moreover, we were not able to analyze patients’ tissues for applying the molecular classification which, however, is able to identify classes of UC with a different molecular and immunological profile (49).

On the other hand, the strength of our study is the prospective measurement of multiple circulating cytokines and inflammatory indexes, standing out against the majority of available data that retrospectively evaluates single biomarkers. Besides the prognostic role, these data could exert a predictive utility, especially integrated into multi-marker classifications, and prospectively validated in future studies in the avelumab maintenance setting, but also in the first line with enfortumab vedotin plus pembrolizumab, following the attempts which have already been made in the pre-treated and first-line settings with ICIs (50–53).

## 5 Conclusions

In the present study, we reported that pre-treatment assessment of cytokines profiles and inflammatory indexes could play a prognostic role in response and survival in mUC patients treated with avelumab as first-line maintenance, and could be integrated into the clinical practice to build multi-parameter classifications and improve treatment tailoring. Our findings support the further in-depth investigation of plasma cytokines and inflammatory indexes as biomarkers for immune phenotype stratification in mUC.

## Data availability statement

The raw data supporting the conclusions of this article will be made available by the authors, without undue reservation.

## Ethics statement

The studies involving humans were approved by Casa Sollievo della Sofferenza. The studies were conducted in accordance with the local legislation and institutional requirements. The participants provided their written informed consent to participate in this study.

## Author contributions

BM: Conceptualization, Data curation, Formal analysis, Funding acquisition, Investigation, Methodology, Project administration, Resources, Software, Supervision, Validation, Visualization, Writing – original draft, Writing – review & editing. GS: Funding acquisition, Resources, Visualization, Writing – review & editing. CC: Formal analysis, Methodology, Visualization, Writing – original draft. GP: Formal analysis, Methodology, Visualization, Writing – original draft. ER: Visualization, Writing – review & editing. MDM: Formal analysis, Methodology, Supervision, Visualization, Writing – review & editing. ADG: Methodology, Supervision, Validation, Visualization, Writing – review & editing. EM: Funding acquisition, Resources, Visualization, Writing – review & editing.
