# Supplementary material for: Prognostic role of circulating cytokines and inflammation indexes for avelumab maintenance in metastatic urothelial carcinoma
Source: Front Immunol. 2024 May 10;15:1401214. doi: 10.3389/fimmu.2024.1401214 (PMC11116647; doi:10.3389/fimmu.2024.1401214)
Supplement: Supplementary file 1 [file Table_1.docx]

**Supplementary Table 1.** Clinical outcomes.

| **Clinical characteristics** | **Number of patients** | ***p* Response** | ***p* PFS** | ***p* OS** |
| --- | --- | --- | --- | --- |
| Outcomes, *n (%)*  Responders  Not-responders | 13 (46.4%)  15 (53.6%) | / | <0.001 | 0.005 |
| Responders classification, *n (%)*  CR  PR  SD | 1 (7.7%)  7 (53.8%)  5 (38.5%) | / | 0.001 | <0.001 |

CR: complete response; OS: overall survival; PFS: probability of progression-free survival; PR: partial response; SD: stable disease

**Supplementary Table 2.** Immune-related adverse events of the included patients

| **irAE** | **Severity grade (CTCAE v.4.0)** | **Rs or N-Rs group** | **Treatment discontinuation** | **p-value** |
| --- | --- | --- | --- | --- |
| Hypothyroidism | 2 | Rs | No | 0.910 |
| Hypothyroidism | 1 | Rs | No | 0.852 |
| Cutaneous rash | 2 | N-Rs | No | 0.836 |
| Hypercalcemia | 2 | Rs | No | 0.170 |
| Hepatic lives enzymes increase | 2 | Rs | No | 0.210 |
| Fever | 1-2 | N-Rs | No | 0.527 |

CTCAE: Common Terminology Criteria for Adverse Events; irAE: immune-related adverse events; N-Rs: not-responders; Rs: responders
